# Assessment of Raw Cow Milk Quality in Smallholder Dairy Farms in Pemba Island Zanzibar, Tanzania

**DOI:** 10.1155/2018/1031726

**Published:** 2018-03-12

**Authors:** S. H. Gwandu, H. E. Nonga, R. H. Mdegela, A. S. Katakweba, T. S. Suleiman, R. Ryoba

**Affiliations:** ^1^Ministry of Education and Vocational Training, P.O. Box 90543, Dar es Salaam, Tanzania; ^2^Department of Veterinary Medicine and Public Health, College of Veterinary Medicine and Biomedical Sciences, Sokoine University of Agriculture, P.O. Box 3021, Chuo Kikuu, Morogoro, Tanzania; ^3^Pest Management Centre, Sokoine University of Agriculture, P.O. Box 3010, Chuo Kikuu, Morogoro, Tanzania; ^4^Department of Veterinary Services, Ministry of Livestock and Fisheries, P.O. Box 295, Zanzibar, Tanzania; ^5^Department of Animal Science and Production, Sokoine University of Agriculture, P.O. Box 3004, Chuo Kikuu, Morogoro, Tanzania

## Abstract

Milk quality depends on the physicochemical characteristics, hygienic standards, and nutritional quality; however, animal husbandry practices, unhygienic harvesting and processing, may affect its quality. A cross-sectional study was conducted between August 2010 and July 2011 to assess the hygiene of cow milk production environment, raw cow milk physicochemical characteristics, and microbial quality and estimate the prevalence of antimicrobial residues using standard methods in Pemba Island. A total of 98 raw cow milk samples from selected smallholder dairy farms were analyzed, and the judgement on the quality used the East African Standards. Generally, the milk production chain was done under the unhygienic condition, and dirty plastic containers were used for collection and storage of milk under room temperature. Some milk samples had abnormal colour (2.1%), abnormal smell (7.1%), and pH below normal (35.7%), clotted on alcohol test (9.2%), and had the specific gravity below normal (13.3%). All the milk samples had mineral contents within the recommended range. Milk samples with butterfat below normal were 29.6%, while 14.3% had total solids below recommended values. The mean total viable count (TVC) of milk container surfaces was 9.7 ± 10.5 log CFU/100 cm^2^, while total coliform count (TCC) was 7.8 ± 8.5 log CFU/100 cm^2^. Up to 55.1% of milk had TVC beyond the recommended levels. The milk mean TVC was 11.02 ± 11.6 log CFU/ml and TCC was 6.7 ± 7.3 log CFU/ml. Up to 26.5% of milk samples had the TCC beyond levels. Results on physicochemical characteristics and nutritional analysis show that the raw cow milk in Pemba Island is of inferior quality. Microbiological results of this study imply heavy contaminations of milk. Antimicrobial residues were detected in 83% of the samples and most of them were from Wete District. Unhygienic milk production chain accelerates microbial contaminations, and antimicrobial residues in milk are a big problem that needs urgent attention from the responsible authority.

## 1. Introduction

The livestock industry in developing countries like Tanzania faces a number of challenges including poor production due to poor animal husbandry, diseases, and poor genetic potential. As compensatory mechanisms, farmers administer a number of veterinary drugs to livestock for prophylaxis and growth promoters [1]. Studies show that the most commonly used drugs are antimicrobials, in particular tetracyclines, penicillins, sulphonamides, and tylosin [1–4]. Because of limited knowledge of possible effects of the drug residues on humans, farmers supply contaminated animal source food for human consumption [4, 5]. Such food sometimes contains high levels of antimicrobial residues which may cause direct health effects like allergic or anaphylactic reactions in humans, direct drug toxicity, and cancer problems [6, 7]. Antibiotic residues pose risks of teratogenicity when administered in the first trimester of pregnancy and permanent discolouration of teeth in infants or children less than 12 years old. More importantly, consuming animal source food containing suboptimal levels of antimicrobials for long periods can increase the rate of development of bacterial resistance and spread of antimicrobial-resistant microorganisms [6, 8, 9]. Also, low concentrations of antimicrobial drug residues create problems in the production of milk products by inhibiting the starter cultures [7, 10].

The occurrences of antibiotic residues in raw cow milk in Tanzania have been reported to range between 0 and 36% [4, 11–15]. The 36% occurrence is very high which implies that the community has been consuming milk with antibiotic residues. Presence of antibiotic residues may affect the physicochemical characteristics of cow milk including abnormal smell as with the case of penicillins. Withdrawal periods, ranging from a few days to a few weeks, are recommended for approved animal drugs. These periods vary according to the drug used, dosage, route of administration, and animal species. Failure to adhere to the recommended periods is the primary cause of illegal levels of veterinary drug residues in animal source food. Among the reasons for the presence of antimicrobial residues in animal source food in Tanzania are limited extension services and poor animal health delivery systems; this situation pushes farmers to buy veterinary drugs from veterinary shops and treat sick animals [1]. When the veterinary drugs are administered by the unprofessional person, problems of incorrect dosage and wrong route of administration are likely to occur [16, 17]. International organizations like Food and Agricultural Organization and World Health Organization (FAO/WHO) and the European Union (EU) have recommended a maximum residue limit (MRL) of some antibiotics like tetracyclines to be 100 *μ*g/l for tetracycline, oxytetracycline, and/or chlortetracycline (singly or in combination) in milk [18–20]. Different antibiotics have also different set residue limits.

Bacterial contamination in milk is another common problem in the dairy setting of developing countries. Bacteria may get access to the milk from the primary source when the animal itself is infected as with the case of mastitic milk. Secondary bacterial contamination in milk is common and is associated with the unhygienic milk production chain [11, 14]. When the milk is contaminated with bacteria, it gets spoiled easily. Bacteria in milk may serve as potential causes of milk-borne diseases in humans. Studies show that up to 90% of all dairy-related diseases are due to pathogenic bacteria like* Brucella abortus*,* Escherichia coli* 0157: H7,* Mycobacterium bovis*,* Campylobacter jejuni, Salmonella* spp,* Clostridium* spp., and* Staphylococcus aureus* [17, 21–23].

In Tanzania, especially in Pemba Island, studies on milk quality are lacking, yet the human population of 406,848 is supplied with locally produced raw cow milk. Previous studies in Tanzania mainland show that majority of the people consume raw untreated cow milk, a practice that puts the public at the risk of milk-borne diseases [4, 24, 25]. Incidences of milk spoilage due to heavy bacterial contamination and poor milk storage facilities are common [4]. The current study was conducted to assess the sanitary practices in milk production chain, evaluate milk quality, and screen for the presence of antimicrobial residues in raw cow milk produced in Pemba Island, Zanzibar, Tanzania.

## 2. Materials and Methods

### 2.1. Study Area, Design, and Animals

This study was conducted in Pemba Island, located in the northernmost of the two islands that make up Zanzibar. The island is situated between 5°13′0′′S and 39°44′0′′E. Pemba Island has an area of 984 km^2^ with the human population of 406,848 [26]. The study design was a cross-sectional study which was conducted between August 2010 and July 2011. The target population was smallholder dairy farmers with lactating cows. In Pemba Island, the smallholder dairy farmers are the sole suppliers of raw cow milk to the community, part of which is consumed as boiled milk or made into milk tea. Nevertheless, a big amount of cow milk is consumed as raw unpasteurized milk or made into different dairy products like fermented milk, butter, cheese, and many other dairy products. For the purpose of this study, raw cow milk samples were collected from four districts namely: Chakechake, Micheweni, Mkoani, and Wete.

The smallholder dairy farmers in Pemba Island keep cattle which are crosses of Friesian, Ayrshire, and Jersey. In some cases, the dairy cattle are managed under semi-intensive management system whereby the animals are grazed on natural pasture and are supplemented with cut grasses and concentrates when animals are back home. A few farmers practice zero grazing whereby the dairy cattle are totally confined and feeding is done indoor. On either of the management systems, the milk production is always low and a cow can produce a maximum of 10 litres of milk per day.

### 2.2. Sampling and Sample Handling

A simple random sampling was adopted to obtain the households of smallholder dairy farms from which milk samples were collected. The sample size of 98 herds of lactating cattle was selected to allow a detection level of 5% with 99% certainty [27]. Before sampling, the observation was done on environmental hygiene, personnel hygiene, milk collection and storage equipment, storage condition, and water used in sanitation and milking procedures. The milk assessment for the smell, colour, any dirty, and cleanliness of containers was done by using standard methods. The smell of milk was assessed after the farmer had opened the lid of the milk container. The colour was examined visually by putting well-stirred milk in the clean glass container as described by Kurwijila et al. [12]. Cleanliness of containers was assessed: “clean” means a container is obviously free from dirty substances, such as abnormal stains and dried milk spillover on surfaces, and its original colour has not been changed due to wear and tear; “satisfactory” means there is no dried milk spillover, there are no abnormal stains that cannot be removed by routine cleaning, and container original colour appears clean, not having been irreversibly changed. “Dirty containers” refer to presence of dried milk spillover, change of container original colour, and presence of abnormal stains that may be removable by cleaning [24].

Sampling for microbiological assessment first involved swab sample collection from the milking equipment and the containers. The containers were swabbed at the bottom round corners using sterile premoistened and dry swabs just before milk was put in the container. An area of 100 cm^2^ was swabbed first with the premoistened swab by rubbing firmly across the area several times in all directions. This was followed by use of the second dry swab which was again rubbed over the same area. Both swabs were immersed in 5 mL buffered peptone water (BPW) in sterile universal bottles and stored in cold storage before analysis. In addition, 50 ml of pooled raw cow milk was sampled from the storage containers after being thoroughly mixed and added to sterile glass bottles and stored in a cool box with ice blocks. The swab and milk samples were transported to Chakechake Veterinary Laboratory for one-week storage at −21°C. Analysis of microbiological contamination was done at Sokoine University of Agriculture microbiology laboratories. Another set of raw cow milk samples (100 ml) was collected for physicochemical assessment which was done according to East African Standards [28].

### 2.3. General Raw Cow Milk Physicochemical Assessment

Determination of pH was done using pH meter, and the pH of 6.6–6.8 for raw cow milk was used as the standard. For the normal milk, the cut-off pH was between 6.6 and 6.8 as recommended in the East African Standards [28]. The alcohol test was done to ascertain the milk acidity, and it involved mixing a 5-ml aliquot of the milk sample with an equal volume of 70% ethanol and examination for the presence of clots. Specific gravity was measured by the use of a lactometer at the standardized temperature of 20°C, and the reference relative density was 1.027–1.032 [28].

Determination of ash content (mineral contents) in raw cow milk was done according to the method of Association of Official Analytical Chemistry [29]. The weight of clean crucible was recorded before the sample was kept in it. Then 5 ml of milk from each sample were poured into a separate crucible and subjected to the high temperature between 500°C and 600°C for 3 hours for ashing. Water and other volatile materials were vaporized and organic substances were burnt to ashes and cooled in desiccators for 3 hours. Thereafter the raw cow milk sample was weighed to determine the concentration of ash present as shown in the formula below. Ash content of milk rarely exceeds 5%.

(1) $$\begin{array}{c} Ash\;\;content\left(\;\%\;\right)=\frac{weight\;\;ashed\;\;sample\;\left(g\right)}{weight\;\;of\;\;milk\;\;sample\;\left(g\right)}\times 100. \end{array}$$

Determination of raw milk fat was done by Gerber method [30]. Ten millilitres of 90% sulphuric acid was added to butyrometer and was mixed with 11 ml of raw milk. Thereafter 1 ml of amyl alcohol was added and rubber stopper was inserted. The butyrometer was shaken carefully until the curd dissolved and no white particles were seen. The butyrometer was placed in the centrifuge with inbuilt heating element pointing towards the centre of the centrifuge. After five minutes of centrifugation at rate of 1100 rpm, the fat column was read from the lowest point of the meniscus on interface of the acid-fat to the 0-mark of the scale, and the milk fat percentage was read [31].

Determination of total solids in raw cow milk was done according to the protocol described by AOAC [29]. Briefly, the raw milk sample was thoroughly mixed and 5 ml was transferred to a preweighed flat bottom dish. After evaporation on steam bath, it was transferred to a hot air oven at 101°C. Dried sample was transferred to a desiccator having silica gel as desiccant. After 1 hour, the dish was weighed and kept in an oven for further drying (~30 minutes). It was again transferred to the desiccator, cooled, and weighed as before. The heating, cooling, and weighing processes were repeated until constant weight was achieved. Total solids content was calculated by the following formula:

(2) $$\begin{array}{c} total\;\;solids\left(\;\%\;\right)=\frac{weight\;\;of\;\;dried\;\;sample\;\left(g\right)}{weight\;\;of\;\;milk\;\;sample\;\left(g\right)}\times 100. \end{array}$$

### 2.4. Microbiological Assessment of Raw Cow Milk

Raw cow milk samples and containers' swabs were assessed for the total viable count (TVC) and total coliform count (TCC) using direct culture methods. Total viable count of microorganisms in raw milk at 37°C was done as per the protocol described by TZS [32] and ISO/FDIS [33]. Microbial colon count on the plates used a protocol described by ISO 7218 [34]. Briefly, tenfold serial dilution of milk sample from 10^−1^ to 10^−10^ in sterile normal saline solution was done, using disposable pipettes. From each dilution, 1 ml of diluted sample was placed in a sterile Petri dish followed by the addition of 20 ml of molten nutrient agar (Oxoid Ltd., Basingstoke, UK), gently shaken, and left to solidify. Incubation was done under the aerobic condition at 37 ± 1°C for 24 ± 3 hours. The microbial colon count on the plates was done with the aid of portable magnifying lens and colonies in the culture plate were countered by using colony counter. Only the plates with 30–300 colonies were considered in calculating the colony forming units (CFU) per ml of sample. According to EAC [28] standards, the TVC should not exceed 5.3 log colony forming units per millilitre (CFU/ml) of raw cow milk.

For total coliform count (TCC), the raw milk samples were thoroughly shaken and then diluted by using the same dilution procedure used for TVC. The samples were inoculated on MacConkey's agar (Oxoid Ltd., Basingstoke, UK) using the spread plate method. The plates were then incubated at 37°C for 36 hours [35]. The number of colonies was recorded using a colony counter. Only the plates with 30–300 colonies were considered in calculating the colony forming units per ml of sample. TCC exceeding 4.5 log CFU/ml in raw cow milk samples means the count is above recommended levels [28].

### 2.5. Assessment of Antimicrobial Residues

Qualitative assessment of antimicrobial residues in raw cow milk was carried out in duplicate using bacterial growth inhibition tests, namely, Delvo SP® test kit (SP mini kit; Delft, Netherlands) that used* Bacillus stearothermophilus* var.* calidolactis*. Briefly, 100 *μ*l of raw milk sample was pipetted into the ampoule with the nutrient tablet. The ampoules were incubated in a water bath with a controlled temperature of 64.0 ± 0.5°C for 3 hours. According to comparisons with the colour of the ampoule containing the control milk sample, a complete purple (blue-violet) colour throughout the whole gel indicated a positive result to antimicrobial residues as they inhibit the growth of the tested strain,* Bacillus stearothermophilus* var.* calidolactis*. In each test, a negative and positive control was included in the analysis.

### 2.6. Data Analysis

Data were entered into Microsoft Excel spreadsheet and analyzed using Epi Info™ version 7 (Centre for Disease Control, Atlanta, USA). The Chi-square (*χ*2) test was used to compare proportions of categorical variables. The proportions of microbial load, antimicrobial residues, and physicochemical properties of milk were compared using Chi-square test at 95% confidence intervals (CI) at the critical probability of *p* < 0.05.

## 3. Results

### 3.1. General Results

**Figure 1:**
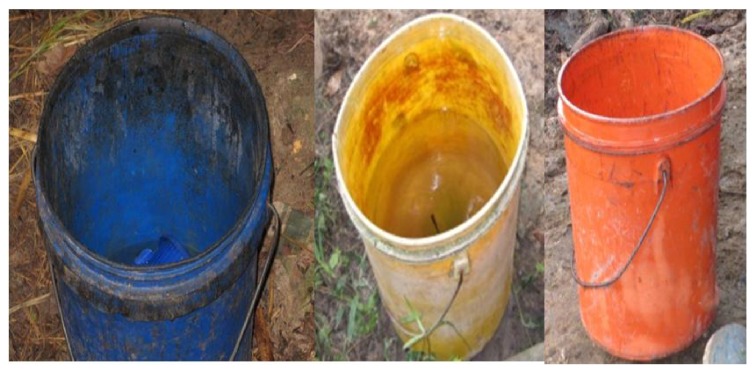
Some plastic containers used for milking and storage of milk. Note that some of the containers look dirty with some obvious dirty stains on the surface to the extent of changing the colour of the container.

A total of 98 households were visited and milk samples collected from Pemba Island. The household distributions in districts were as follows: Chakechake, 35 households; Micheweni, 15; Mkoani, 15; and Wete, 33. On observation of environmental hygiene in animal houses and milking environment, it was found that some of the areas (68.4%) appeared dirty. The floor in most of the animal houses had potholes and cow dung was scattered all over the milking areas. The personnel involved in milking reported washing their hands with cold water before milking. Up to 90.8% of the farmers washed the cow udder with cold water. Some of the milkers (42.9%) reported wiping the udder to dry before milking using a piece of cloth (towel) which was used for all milking cows. Milking in study farms was done by hands within the animal houses. Plastic containers were the commonly (95.6%) used equipment during milking and they appeared dirty in most of the households visited (Figure 1). There was a significant difference between districts on cleaning containers (*p* = 0.0000). Most of the milk containers (80%) had a lid. No cold storage was observed. Immediately after milking, the milk was sold to customers who were mostly neighbours, restaurant/milk kiosk owners, and vendors.

### 3.2. Physicochemical Parameters of Raw Cow Milk

Physicochemical parameter results of raw cow milk samples are shown in Table 1. In all cases, the East African Standards were used as references to milk quality recommended levels. Of all the milk assessed, 2.1% had abnormal colour while 7.1% had the abnormal smell. It was found that the milk mean pH was 6.6 ± 0.109 (range 6.3–6.9); the samples that had pH below normal value were 35 (35.7%), but on alcohol test, only 9 (9.2%) were clotted. Lactometer reading indicated that the mean milk-specific gravity was 30.5 ± 3.8 (range 22–37), and 13 (13.3%) of the tested milk samples had the specific gravity below the normal level of 1.026. The mean ash (mineral) contents were 0.7 ± 0.2% (range 0.4–1.805%), and all the milk samples tested (*n* = 98) had the levels within the normal range of 0.4–5%. The mean milk butterfat was 3.8 ± 1.4% (range 0.8–9%), and 29 (29.6%) of the samples had butterfat below normal. The differences in milk butterfat levels between districts were statistically significant (*p* = 0.005). It was also observed that the mean total solids in milk were 12.8 ± 2.04% (range = 9.3–23.9%), and 14 (14.3%) of the samples tested were found to have total solids below normal recommended values.

### 3.3. Total Viable Count of Microbes

Microbiological results showed that the mean TVC of swabs was 9.7 ± 10.5 log CFU/100 cm^2^ (range 1.5–11.4 log CFU/100 cm^2^). The mean TCC of swabs was 7.8 ± 8.5 log CFU/100 cm^2^ (range 1.5–9.3 log CFU/100 cm^2^). Results of TVC in milk showed that 54 (55.1%) of the milk samples assessed had the TVC beyond the recommended level of 5.3 log CFU/ml. The milk mean TVC was 11.02 ± 11.6 log CFU/ml (range 2.5–12.4) and the mean TCC was 6.7 ± 7.3 log CFU/ml (range 1.5–8.1 log CFU/100 cm^2^). Results of TCC in milk showed that 26 (26.5%) of the milk samples assessed had the TCC beyond the recommended level of 4.7 log CFU/ml. District-wise, the mean TVC and TCC (log CFU/ml) are shown in Table 2. It was observed that Micheweni and Mkoani districts had the highest means of TVC while Wete milk samples had the highest TCC compared to the rest. In all these cases, the differences in terms of TVC and TCC between districts were not statistically significant (*p* > 0.05).

### 3.4. Antimicrobial Residues in Milk Produced in Pemba Island

Qualitative antimicrobial residue laboratory results indicated that 83% of the screened raw cow milk was positive to antimicrobial residues test by Delvo SP test assay. Most of the positive milk samples (26%) were those from Wete District (Table 3).

## 4. Discussion

Generally, the milking environment was found to predispose the raw cow milk to microbial contaminations. The areas appeared rather dirty; the use of cold water in the washing of the udder and poorly washed plastic containers may have contributed to high microbial contaminations that were observed. The plastic milk containers always appeared dirty and some of them had their colour changed because of insufficient cleaning (Figure 1). This enhanced accumulation of some fats and other sticky materials which otherwise acted as media for microbial growth [17, 36]. Indeed, the microbiological assessment of milk containers revealed extremely high mean TVC (9.7 ± 10.5 log CFU/100 cm^2^) suggestive of heavy contaminations.

Interestingly, the mean TCC of swabs was 7.8 ± 8.5 log CFU/100 cm^2^ further indicating poor hygiene in containers. Presence of coliform bacteria in food surfaces does not necessarily indicate faecal contaminations but may indicate that the hygiene status of the preparation and processing is poor [37]. It was observed that milk containers were being washed with cold water collected from wells which potentially serve as sources of coliform bacteria as previously reported by Kivaria et al. [17]. Therefore, with such high rates of milk container contamination, they will always serve as sources of microbial contaminations in milk. The use of one towel in drying the udder may spread the microbes from one cow to the other and may not only become the sources of milk contaminations but enhance the spread of bacteria that cause diseases like mastitis in cows. It is recommended that farmers have to be educated on hygienic practices of milking and milk handling along the production chain so as to minimize possibilities of microbial contaminations which otherwise are responsible for spoilage and the cause of milk-borne diseases in humans.

It was found that a small proportion of raw cow milk had abnormal colour and smell which may suggest contaminations of different kinds like cow dugs, hairs, other dirty, and some cases of animal diseases like mastitis. Nevertheless, the abnormal smell may be due to spoiled milk, dirty containers, type of feeds, drugs, acaricides, and animal diseases like pyometra. Good quality milk is supposed to be free from any objectionable things like smell and colour. According to East African Standards [28], the recommended raw cow milk pH is 6.6–6.8 which can withstand the boiling temperature. During this study, some milk samples (35.7%) had pH below normal values which implied that they were acidic. Interestingly, only 9 out of 35 samples with low pH were clotted on the alcohol test. There are many factors which can make the raw cow milk acidic, but the major one is poor storage under room temperature which accelerates microbial activities on lactose which is normally converted to lactic acid [14]. Sometimes, low pH of milk may be due to presence of high levels of casein, acid phosphates and citrates, albumin, globulin, and carbon-dioxide [38, 39]. Availability of power supply is a problem in Tanzania, especially in rural and periurban areas; therefore it was difficult to store the milk under refrigeration temperature. Nevertheless, it is important that farmers have to be educated on proper storage of milk so as to avoid unnecessary losses because of spoilage.

The lactometer reading indicated that the mean raw cow milk-specific gravity (30.5 ± 3.8) was within the recommended level. However, some (13.3%) of the tested raw cow milk had specific gravity below 1.026. Indeed, all the milk samples that had low specific gravity were also found to have low total solids than the recommended values. Many factors are known to cause low specific gravity of raw cow milk which ranges from animal factors (breed, stage of lactation), diseases and symptoms like mastitis and fever, type of animal feed, and adulterations like the addition of water [40]. All the milk samples tested were from crossbred dairy cows which were zero grazed. Therefore either of the above-stated factors may contribute to the low specific gravity of the milk. All the milk samples that had low specific gravity had also low total solids.

Total solids in raw cow milk are an important indicator of nutritional value as it determines the levels of carbohydrates, fats, proteins, vitamins, and minerals. In the raw cow, they are supposed to range between 10 and 14%. However, total solids of raw cow milk may be influenced by many factors that include breeds of cattle, stage of lactation, season, feeds, age, health, and physiological status of the animal [38, 41]. This study established that the mean total solids in raw cow milk were 12.8 ± 2.04% (range 9.3–23.9%). A total of 14 (14.3%) samples had total solids below normal recommended values for raw cow milk. Other studies elsewhere also had similar observations on total solids in raw cow milk [14, 42–44]. Nevertheless, all the milk samples tested had the levels of mineral contents within the normal range of 0.4–5% while 29.6% of the samples had butterfat below normal.

The study further established that the raw cow milk produced in Pemba was heavily contaminated by microbes. Results of TVC in milk showed that 54 (55.1%) of the milk samples assessed had the TVC beyond the recommended level of 5.3 log CFU/ml of EAC standards [28] and the count established was up to 12.4 log CFU/ml. Microbial contaminations in raw cow milk may originate from the animal itself when is infected, but mostly microbes enter the milk along with the handling chain. During this study, it was established that milking environment, milking process, milk handling, and storage were done under poor sanitary conditions. This may be the main reason that potentiated the high microbial count in the milk. Other studies in Tanzania reported that unhygienic practices along the milk value chain predisposed milk to high bacterial load [4, 24, 25]. Elsewhere in Africa and Asia also high raw milk contamination rates and the possible factors for contaminations reported are more or less the same as the current study [45, 46].

It was further found that the mean TCC in milk was 6.7 ± 7.3 log CFU/ml with the highest count summing to 8.1 log CFU/ml. This heavy contamination was observed in many samples and the results indicated that 26.5% of the milk samples had the coliform count beyond the recommended level of 4.7 log CFU/ml [28]. The high coliform counts are especially associated with the level of hygiene and are an indication of faecal contamination from animals, humans, or environmental materials [42, 47]. High coliform counts imply also that the risk of pathogenic enterobacteria in the samples is high [48, 49]. Coliforms can rapidly build up in the moist residues on the milking equipment which potentially serves as a source of contamination for the milk [50]. It is therefore important that the raw cow milk should be harvested hygienically and kept in clean containers under refrigeration temperatures immediately after milking process. This good practice tends to minimize multiplication of microbes in milk between milking at the farm and transportation to the customers [4]. The finding of our study is in line with other studies in Tanzania and elsewhere [4, 24, 25, 42].

Of great interest, qualitative antimicrobial residue analysis indicated an exceptionally high (82.7%) rate of raw cow milk contaminations compared to the previous similar studies in Tanzania [4, 11, 12, 14, 15, 17, 51, 52]. With such high rates of antimicrobial residues, it is implied that there are indiscriminate uses of antimicrobials in dairy cattle and the farmers do not abide by the withdrawal period. Antibiotic residues in animal source food are the potential causes of antibiotic resistance in bacteria. Other studies elsewhere have also reported high levels of antimicrobial residues in milk [8, 53–55]. The differences in antibiotic residue levels in milk may be due to different production and monitoring systems and laboratory analysis used in testing of the antimicrobial residues.

The very high rates of antimicrobial residues in milk observed in the current study may also be due to the limitation of the test method used, Delvo test. The test is constrained by the presence of natural inhibitors in milk such as lactoferrin, lactoperoxidase, lysozyme, and N-acetyl-ß-D-glucosaminidase which all have antimicrobial properties with potentials of inhibiting the growth of test bacteria,* Bacillus stearothermophilus* var.* calidolactis* which was used in Delvo test [56]. Therefore, it is recommended that before concluding with certainty on the rates of antimicrobial residues in raw cow milk from Pemba, confirmatory methods are to be used. Nevertheless, the screening results reported in this study imply that there is poor regulation enforcement by Tanzania Food and Drug Authority (TFDA) on routine milk assessment for antimicrobial residue. Farmers need more education on effects caused by drug residues in food of animal origin and the importance of controlled antimicrobial uses in livestock production.

## Figures and Tables

**Table 1:** Physicochemical assessment results of raw cow milk in Pemba.

Physicochemical parameter	Category	Number (%) of milk samples from different districts				Total number (%) of milk samples (*n* = 98)
		Chakechake *n* = 35	Micheweni *n* = 15	Mkoani *n* = 15	Wete *n* = 33	
Colour	Normal milky	34 (97.1)	14 (93.3)	15 (100)	33 (100)	96 (97.9)
	Abnormal	1 (2.9)	1 (6.7)	0 (0.0)	0 (0.0)	2 (2.1)

Smell	Normal milk smell	33 (94.3)	12 (80.0)	15 (100)	31 (93.9)	91 (92.9)
	Bad	2 (5.7)	2 (20.0)	0 (0.0)	2 (6.1)	7 (7.1)

Clotting on alcohol test	No	31 (88.6)	14 (93.3)	13 (86.7)	31 (88.6)	89 (90.8)
	Yes	4 (11.4)	1 (6.7)	2 (13.3)	2 (11.4)	9 (9.2)

pH	<6.6	8 (22.9)	6 (40.6)	4 (26.7)	17 (51.5)	35 (35.7)
	Normal (6.6–6.8)	26 (74.3)	9 (60.0)	10 (66.7)	15 (45.5)	60 (61.2)
	>6.8	1 (2.9)	0 (0.0)	1 (6.7)	1 (3.0)	3 (3.1)

Specific gravity (g/ml)	<1.027	13 (37.1)	5 (33.3)	8 (53.4)	8 (24.2)	34 (34.7)
	Normal (1.027–1.032)	16 (45.7)	8 (53.4)	7 (46.6)	24 (72.7)	55 (56.1)
	>1.032	6 (17.1)	2 (13.3)	0 (0.0)	1 (3.1)	9 (9.2)

Ash contents	Normal (0.4–5%)	35 (100)	15 (100)	15 (100)	33 (100)	98 (100)

Butterfat	Normal (3.25–8.5%)	22 (62.9)	13 (86.7)	6 (40.0)	28 (84.8)	69 (70.4)
	<3.25%	13 (37.1)	2 (13.3)	9 (60.0)	5 (15.2)	29 (29.6)
	>8.5%	0 (0.0)	0 (0.0)	0 (0.0)	0 (0.0)	0 (0.0)

Total solids	Normal (10–14%)	30 (85.7)	13 (86.7)	10 (66.7)	31 (93.9)	84 (85.7)
	<10	5 (14.3)	2 (13.3)	5 (33.3)	2 (6.1)	14 (14.3)
	>14%	0 (0.0)	0 (0.0)	0 (0.0)	0 (0.0)	0 (0.0)

**Table 2:** Types of microbial contamination in milk from different districts of Pemba.

Microbial category	District	Mean (±stdv) count (log CFU/ml)	Range
Total viable count (TVC)	Chakechake (*n* = 35)	10.8 ± 11.1	2.5–11.7
	Micheweni (15)	11.5 ± 11.9	2.5–12.3
	Mkoani (*n* = 15)	11.2 ± 11.8	3.6–12.3
	Wete (33)	10.8 ± 11.5	2.6–12.2

Total coliform count (TCC)	Chakechake (*n* = 14)	5.8 ± 6.3	1.5–6.7
	Micheweni (*n* = 9)	7.2 ± 7.6	1.5–8.1
	Mkoani (*n* = 5)	6.3 ± 6.5	4.9–6.9
	Wete (*n* = 20)	6.5 ± 6.6	1.8–7.1

**Table 3:** Antimicrobial residues in milk per district in Pemba Island.

District	Number of positive milk samples	Percentage of positive milk samples
Chakechake	30	31
Micheweni	14	14
Mkoani	12	12
Wete	25	26
Total	81	82.7

## References

[1] Katakweba A. A. S., Mtambo M. M. A., Olsen J. E., Muhairwa A. P. (2012). Awareness of human health risks associated with the use of antibiotics among livestock keepers and factors that contribute to selection of antibiotic resistance bacteria within livestock in Tanzania. *Livestock Research for Rural Development*.

[2] Midenge B. Y. (2011). *Awareness on recommended veterinary drugs withdrawal period among small scale dairy cattle farmers in Kinondoni Municipality*.

[3] Kimera Z. I. (2013). *Evaluation of oxytetracycline residues in beef in Kilosa district Tanzania*.

[4] Bukuku J. N., Nonga H. E., Mtambo M. M. A. (2015). Assessment of raw milk quality and stakeholders’ awareness on milk-borne health risks in Arusha City and Meru District, Tanzania. *Tropical Animal Health and Production*.

[5] Nonga H. E., Simon C., Karimuribo E. D., Mdegela R. H. (2009). Assessment of antimicrobial usage and residues in commercial chicken eggs from smallholder poultry keepers in morogoro municipality, Tanzania. *Zoonoses and Public Health*.

[6] Layada S., Benouareth D., Coucke W., Andjelkovic M. (2016). Assessment of antibiotic residues in commercial and farm milk collected in the region of Guelma (Algeria). *International Journal of Food Contamination*.

[7] Petrović J. M., Katić V. R., Bugarski D. D. (2008). Comparative Examination of the Analysis of *β*-Lactam Antibiotic Residues in Milk by Enzyme, Receptor–Enzyme, and Inhibition Procedures. *Food Analytical Methods*.

[8] Aalipour F., Mirlohi M., Jalali M. (2013). Prevalence of antibiotic residues in commercial milk and its variation by season and thermal processing methods. *International Journal of Environmental Health Engineering*.

[9] Granados-Chinchilla F., Rodríguez C. (2017). Tetracyclines in Food and Feedingstuffs: From Regulation to Analytical Methods, Bacterial Resistance, and Environmental and Health Implications. *Journal of Analytical Methods in Chemistry*.

[10] Stead S. L., Ashwin H., Richmond S. F. (2008). Evaluation and validation according to international standards of the Delvotest® SP-NT screening assay for antimicrobial drugs in milk. *International Dairy Journal*.

[11] Karimuribo E. D., Kusiluka L. J., Mdegela R. H., Kapaga A. M., Sindato C., Kambarage D. M. (2005). Studies on mastitis, milk quality and health risks associated with consumption of milk from pastoral herds in Dodoma and Morogoro regions, Tanzania. *Journal of Veterinary Science*.

[12] Kurwijila L. R., Omore A., Staal S. (2009). Dairy Sub Sector Development Strategy, East Africa Regional Initiatives in Value Chains. *Lesson from on- going R & D initiatives in Dairy value chains, Rural Livelihood Development Company. Version Board*.

[13] Kaale E., Chambuso M., Kitwala J. (2008). Analysis of residual oxytetracycline in fresh milk using polymer reversed-phase column. *Food Chemistry*.

[14] Mdegela R. H., Ryoba R., Karimuribo E. D. (2009). Prevalence of clinical and subclinical mastitis and quality of milk on smallholder dairy farms in Tanzania. *Journal of the South African Veterinary Association*.

[15] Rwehumbiza J. M., Ryoba R., Karimuribo E. D. TVA Conference on Contribution of the Veterinary Profession to the Improvement of Human Health.

[16] Shitandi A. (2004). *Risk factors and control strategies of antibiotic residues in milk at farm level in Kenya*.

[17] Kivaria F. M., Noordhuizen J. P. T. M., Kapaga A. M. (2006). Evaluation of the hygienic quality and associated public health hazards of raw milk marketed by smallholder dairy producers in the Dar es Salaam region, Tanzania. *Tropical Animal Health and Production*.

[18] Applegren L., Arnold D., Boisseau J., Boobis A., Ellis R., Livingston R. (1999). Evaluation of certain veterinary drug residues in food. *Fiftieth Report of the Joint FAO/WHO Expert Committee on Food Additives*.

[19] EU (European Commission) (2009). On pharmacologically active substances and their classification regarding maximum residue limits in foodstuffs of animal origin. *Commission Regulation (EU)*.

[20] Codex Alimentarius Commission (CAC) Maximum Residue Limits for Veterinary Drugs in Foods.

[21] Shirima G. M., Kazwala R. R., Kambarage D. M. (2003). Prevalence of bovine tuberculosis in cattle in different farming systems in the eastern zone of Tanzania. *Preventive Veterinary Medicine*.

[22] Donkor E., Aning K., Quaye J. (2010). Bacterial contaminations of informally marketed raw milk in Ghana. *Ghana Medical Journal*.

[23] Hagstad H. V., Hubbert W. T. (1986). *Food quality control: Food of Animal Origin*.

[24] Nonga H. E., Ngowi H. A., Mdegela R. H. (2015). Survey of physicochemical characteristics and microbial contamination in selected food locally vended in Morogoro Municipality, Tanzania Microbiology. *BMC Research Notes*.

[25] Kanyeka H. B. (2014). *Assessment of microbial quality of raw cow’s milk and antimicrobial susceptibility of selected milk-borne bacteria in Kilosa and Mvomero districts, Tanzania*.

[26] PHCT (2012). The 2012 Population and Housing Census of Tanzania General Report.

[27] Canon R. M., Roe R. T. (1986). *Livestock disease surveys: A field manual for veterinarians*.

[28] East African Community (EAC) (2006). *East African Standard, Raw cow Milk Specification*.

[29] AOAC (1990). *Official Methods of Analysis*.

[30] James C. S. (1995). Determination of the fat content of dairy products by the Gerber Method. *Analytical Chemistry of Food*.

[31] Marshall R. T. (1992). *Standard Methods for the determination of Dairy Products*.

[32] TZS (Tanzania Bureau of Standards) (2007). Microbiology of food and animal feeding stuffs-method for enumeration of microorganisms—colony count technique at 30°C. *Tanzania Standards Specification*.

[33] ISO/FDIS (2001). *Milk and milk products—general guidance for the preparation of samples, initial suspensions and decimal dilutions for microbiological examination*.

[34] ISO 7218:2007, “(International Standards Organisation): Microbiology of food and animal feeding stuffs- general requirements and guidance for microbiological examination”, No. 7218; pp. 8–15, 2007

[35] Harley J. P. (2013). *Laboratory Exercises in Microbiology*.

[36] Hayes M. C., Ralyea R. D., Murphy S. C., Carey N. R., Scarlett J. M., Boor K. J. (2001). Identification and characterization of elevated microbial counts in bulk tank raw milk. *Journal of Dairy Science*.

[37] Simforian E., Nonga H. E., Ndabikunze B. K. (2015). Assessment of microbiological quality of raw fruit juice vended in dar es salaam city, Tanzania. *Food Control*.

[38] Vishweshwar S. K., Krishnaiah N. (2005). *Quality Control of Milk And Processing*.

[39] Bille P. G., Haradoeb B. R., Shigwedha N. (2009). Evaluation of chemical and bacteriological quality of raw milk from Neudamm dairy farm in Namibia. *African Journal of Food, Agriculture, Nutrition and Development*.

[40] Pandey G. S., Voskuil G. C. S. (2011). *Manual on Milk Safety, Quality and Hygiene*.

[41] O’Connor C. B. (1995). *Rural dairy Technology ILRI Training Manual I*.

[42] Omore A. O., Arimi S. M., Kang'ethe E. K., McDermott J. J., Staal S. J. Analysis of milk-borne public health risks in milk markets in Kenya.

[43] Javaid S. B., Gadahi J. A., Khaskeli M., Bhutto M. B., Kumbher S., Panhwar A. H. (2009). Physical and chemical quality of market milk sold at tandojam, Pakistan. *Pakistan Veterinary Journal*.

[44] Gemechu T., Fekadu B., Eshetu M. (2015). Physical and chemical quality of raw cow’s milk produced and marketed in Shashemene Town, Southern Ethiopia. *ISABB-Journal of Food and Agricultural Science*.

[45] A Ogot H., Ochuodho H. O., Machoka R. Microbial analysis of raw and boiled milk sold at Baraton center in Nandi County, Kenya.

[46] Kaindi D. W. M., Schelling E., Wangoh J., Imungi J. K., Farah Z., Meile L. (2011). Microbiological quality of raw camel milk across the Kenyan market chain , Food 5. Global Science Books. *Food 5. Global Science Books, Special Issue 1*.

[47] Robinson R. K. (2002). *Dairy Microbiology Handbook: The Microbiology of Milk and Milk Products*.

[48] Chye F. Y., Abdullah A., Ayob M. K. (2004). Bacteriological quality and safety of raw milk in Malaysia. *Food Microbiology*.

[49] Mennane Z., Ouhssine M., Khedid K., ELyachioui M. (2007). Hygienic quality of raw cows milk feeding from domestic waste in tow regions in Morocco. *International Journal of Agriculture and Biology*.

[50] Omore A. O., Arimi S. M., Kang'ethe E. K., McDermott J. J., Staal S. J., Ouma E. A. (2001). Assessing and management milk-borne health risks for the benefit of consumers in Kenya. *Smallholder Dairy (R and D) Project (SDP)*.

[51] Kurwijila L. R., Omore A., Staal S., Mdoe N. S. Y. (2006). Investigation of the risk of exposure to antimicrobial residues present in marketed milk in Tanzania. *Journal of Food Protection*.

[52] Mhozya M. (2017). *Assessment of Brucellaabortus and antimicrobial residues in raw milk in Bukombe, Tanzania*.

[53] Salman A. M., ElNasri H. A., Osman I. A. M. (2012). Detection of antibiotic residues in milk using delvotest kit and the disc assay methods in Khartoum State, Sudan. *Journal of Veterinary Medicine and Animal Production*.

[54] Ahlberg S., Korhonen H., Lindfors E., Kangethe E. (2016). Analysis of antibiotic residues in milk from smallholder farms in Kenya. *African Journal of Dairy Farming and Milk Production*.

[55] Orwa J. D., Matofari J. W., Muliro P. S., Lamuka P. (2017). Assessment of sulphonamides and tetracyclines antibiotic residue contaminants in rural and peri urban dairy value chains in Kenya. *International Journal of Food Contamination*.

[56] Kang J. H., Jin J. H., Kondo F. (2005). False-positive outcome and drug residue in milk samples over withdrawal times. *Journal of Dairy Science*.

